# Nierentransplantation: Der Einfluss von biologischem Geschlecht und dem kulturellen Geschlechtsbegriff auf die transplantatbezogenen und patientenrelevanten Ergebnisse

**DOI:** 10.1007/s00120-026-02779-7

**Published:** 2026-01-30

**Authors:** Laila Schneidewind

**Affiliations:** Universitätsklinik für Urologie, Inselspital Bern, Freiburgstr. 37, 3010 Bern, Schweiz

## Originalpublikation

Jayanti S, Beruni NA, Chui JN, Deng D, Liang A, Chong AS, Craig JC, Foster B, Howell M, Kim S, Mannon RB, Sapir-Pichhadze R, Scholes-Robertson NJ, Strauss AT, Jaure A, West L, Cooper TE, Wong G. Sex and gender as predictors for allograft and patient-relevant outcomes after kidney transplantation. Cochrane Database Syst Rev. 2024 Dec 19;12(12):CD014966.

## Hintergrund.

Das Geschlecht als biologisches Konstrukt (im Folgenden biologisches Geschlecht) und das Geschlecht (im Folgenden kultureller Geschlechtsbegriff), definiert als die kulturellen Einstellungen und Verhaltensweisen, die von der Gesellschaft zugeschrieben werden, können mit dem Verlust von Allotransplantaten, Tod, Krebs und Abstoßung in Verbindung gebracht werden. Andere Faktoren, wie das Alter des Empfängers und das biologische Geschlecht des Spenders, können den Zusammenhang zwischen den beiden hier genannten Geschlechtsaspekten und Ergebnissen nach der Transplantation verändern.

## Ziele.

Wir haben versucht, die prognostischen Wirkungen des biologischen Geschlechts des Empfängers und, getrennt davon, des kulturellen Geschlechtsbegriffs als unabhängige Prädiktoren für Transplantatverlust, Tod, Krebs und Allotransplantatabstoßung nach Nieren- oder gleichzeitiger Pankreas-Nieren-Transplantation (SPK) zu bewerten. Wir wollten diesen prognostischen Effekt bewerten, indem wir die Beziehung zwischen dem biologischen Geschlecht des Empfängers oder dem kulturellen Geschlechtsbegriff sowie den Endpunkten nach der Transplantation definierten, die Gründe für die Unterschiede zwischen den biologischen Geschlechtern sowie den kulturellen Geschlechtsbegriffen ermittelten und dann das Ausmaß dieser Beziehung quantifizierten.

## Suchmethodik.

Wir durchsuchten die Datenbanken MEDLINE und EMBASE von Beginn an bis zum 12. April 2023, wobei wir uns an den Cochrane Kidney and Transplant Information Specialist wandten und für diese Überprüfung relevante Suchbegriffe verwendeten.

## Auswahlkriterien.

Kohorten‑, Fall-Kontroll- oder Querschnittstudien wurden einbezogen, wenn biologisches Geschlecht oder kultureller Geschlechtsbegriff die primäre Exposition waren und klar definiert wurden. Die Studien sollten sich auf die von uns definierten Endpunkte nach der Transplantation konzentrieren. Das biologische Geschlecht wurde definiert als die chromosomalen, gonadalen und anatomischen Merkmale, die mit dem biologischen Geschlecht assoziiert werden, und wir verwendeten die Begriffe „männlich“ und „weiblich“. Der kulturelle Geschlechtsbegriff wurde definiert als die Einstellungen und Verhaltensweisen, die eine bestimmte Kultur mit dem biologischen Geschlecht einer Person in Verbindung bringt, und wir verwendeten die Begriffe „Männer“ und „Frauen“.

## Datensammlung und Datenanalyse.

Zwei Autoren bewerteten unabhängig voneinander die Referenzen auf ihre Eignung, extrahierten die Daten und bewerteten das Risiko einer Verzerrung anhand des QUIPS-Tools (Quality in Prognosis Studies). Wann immer es angebracht war, haben wir ein Random-effekts-Modell für Metaanalysen verwendet, um den mittleren Unterschied in den Endpunkten zu schätzen. Zu den Endpunkten von Interesse gehörten die „standardised outcomes“ in Nephrology-Kidney Transplant (SONG-Tx), Allotransplantatverlust, Tod, Krebs (allgemeine Inzidenz und ortsspezifisch) und akute oder chronische Transplantatabstoßung.

## Hauptergebnisse.

Berücksichtigt wurden 53 Studien (2.144.613; Spanne 59–407.963 Patient:innen), die zwischen 1990 und 2023 durchgeführt worden sind. 16 Studien wurden in Nord- und Südamerika, 12 in Europa, 11 im westlichen Pazifik, 4 im östlichen Mittelmeerraum, 3 in Afrika, 2 in Südostasien und 5 parallel in mehreren Regionen durchgeführt. Mit einer Ausnahme konzentrierten sich alle Studien auf das biologische Geschlecht und nicht auf den kulturellen Geschlechtsbegriff als primäres Interessensgebiet.

Der Anteil der Männer lag bei 54 %. 49 Studien umfassten Empfänger von Nierentransplantaten und 4 Studien umfassten Empfänger von SPK-Transplantaten. 24 Studien umfassten Erwachsene und Kinder, 25 Studien nur Erwachsene und 4 Studien ausschließlich Kinder.

In die Metaanalysen wurden Daten aus 33 Studien einbezogen. In 6 dieser Studien wurde die Wirkung des biologischen Geschlechts des Empfängers auf den Verlust von Nierentransplantaten anhand unbereinigter Hazard Ratios (HR) untersucht. Die anderen Studien gaben Risikoverhältnisse (RR) für die vordefinierten Endpunkte an. Die Entscheidung, die Metaanalysen auf nicht bereinigte Schätzungen zu beschränken, ergab sich insbesondere aus den unterschiedlichen Methoden der Kovariatenbereinigung in den einzelnen Studien, da es keinen gemeinsamen Satz bereinigter Variablen gab. Nur in 3 Studien wurde die modifizierende Wirkung des Alters des Empfängers auf den Transplantatverlust oder den Tod berücksichtigt, was für die Bewertung der geschlechtsspezifischen Unterschiede bei den Endpunkten nach der Transplantation wahrscheinlich entscheidend ist. In keiner Studie wurde die modifizierende Wirkung des Alters des Empfängers auf die Krebsinzidenz oder das Risiko der Abstoßung von Allotransplantaten untersucht.

In einer Evidenz von niedriger Vertrauenswürdigkeit macht es im Vergleich zu männlichen Empfängern keinen oder nur einen geringen Unterschied, ob eine Frau eine Nierentransplantation erhält oder nicht (7 Studien, 5843 Patienten: RR 0,91; 95 %-Konfidenzintervall [KI] 0,73 bis 1,12; I^2^ = 73 %). Dies wurde auch in Studien beobachtet, die Analysen von der Zeit bis zum Ereignis enthielten (6 Studien, 238.937 Patienten; HR 1,07; 95 %-KI, 0,95 bis 1,20; I^2^ = 44 %).

Zwei kürzlich durchgeführte große registergestützte Kohortenstudien, in denen die modifizierenden Wirkungen des biologischen Spendergeschlechts und des Alters des Empfängers berücksichtigt wurden, zeigten, dass weibliche Empfänger unter 45 Jahren signifikant höhere Transplantatverlustraten aufwiesen als altersgleiche männliche Empfänger mit einem männlichen Spender. Im Gegensatz dazu hatten weibliche Empfänger, die 60 Jahre und älter waren, unabhängig vom Geschlecht des Spenders niedrigere Transplantatverlustraten als altersgleiche männliche Empfänger. Im Vergleich zu männlichen Empfängern macht die Tatsache, dass sie weiblich sind, bis zu 30 Jahre nach der Transplantation keinen oder nur einen geringen Unterschied bei der Sterblichkeit aus; die Evidenz ist jedoch sehr unsicher (13 Studien; 60.818 Patienten: RR 0,94; 95 %-KI 0,81 bis 1,09; I^2^ = 92 %).

Studien, die die modifizierenden Wirkungen des Alters des Empfängers und des biologischen Geschlechts des Spenders berücksichtigten, ergaben, dass weibliche Empfänger bei einem männlichen Spender ein höheres Sterberisiko hatten als Männer (mit männlichem Spender) unter 45 Jahren. Im Vergleich zu männlichen Empfängern macht die Tatsache, dass sie weiblich sind, möglicherweise nur einen geringen oder gar keinen Unterschied in der Krebsinzidenz bis zu 20 Jahre nach der Transplantation; die Evidenz ist jedoch sehr unsicher (7 Studien; 25.076 Patienten; RR 0,84; 95 %-KI 0,70 bis 1,01; I^2^ = 60 %). Im Vergleich zu männlichen Empfängern macht die Tatsache, dass sie weiblich sind, keinen oder nur einen geringen Unterschied in der Häufigkeit der akuten und chronischen Abstoßung von Nierentransplantaten bis zu 15 Jahre nach der Transplantation (9 Studien; 6158 Patienten: RR 0,89; 95 %-KI 0,75 bis 1,05; I^2^ = 54 %; Evidenz von niedriger Vertrauenswürdigkeit).

Eine Studie untersuchte den kulturellen Geschlechtsbegriff und berichtete, dass Frauen im Vergleich zu Männern in Gebieten mit hohem (HR 0,71; 95 %-KI 0,59 bis 0,87) und mittlerem Einkommen (HR 0,82; 95 %-KI 0,74 bis 0,92) eine bessere 5‑Jahres-Überlebensrate aufwiesen, während es in Gebieten mit niedrigem Einkommen keinen Unterschied gab (HR 0,85; 95 %-KI 0,72 bis 1,01).

Es bestand erhebliche Unsicherheit hinsichtlich eines Zusammenhangs zwischen biologischem Geschlecht oder kulturellem Geschlechtsbegriff und patientenrelevanten Endpunkten nach der Transplantation. Dies war in erster Linie auf die klinische und methodische Heterogenität zurückzuführen. Die beobachtete klinische Heterogenität zwischen den Studien könnte auf unterschiedliche Patientenmerkmale innerhalb der Stichprobenpopulationen zurückzuführen sein. Da nur wenige nach biologischem Geschlecht aufgeschlüsselte demografische Daten zur Verfügung standen, konnte diese Heterogenität nicht weiter untersucht werden. Zu den Faktoren, die zu diesem Ergebnis beitragen, gehören jedoch möglicherweise das Alter des Empfängers, das Alter des Spenders, der Spendertyp und der kulturelle Geschlechtsbegriff.

Methodische Heterogenität wurde durch die austauschbare Verwendung von biologischem Geschlecht und kulturellem Geschlechtsbegriff, die Fehlklassifizierung von Endpunkten, die Verwendung unterschiedlicher Maße für Wirkungen, inkonsistente Kovariatenprofile und die Nichtberücksichtigung wichtiger Effektmodifikationen festgestellt.

## Schlussfolgerung der Autoren.

Die Evidenz von sehr niedriger bis niedriger Vertrauenswürdigkeit deutet darauf hin, dass es keine Unterschiede zwischen männlichen und weiblichen Empfängern von Nieren- und Pankreastransplantaten in Bezug auf das Überleben von Nieren- und Pankreastransplantaten, das Überleben der Patienten, Krebs und akute sowie chronische Abstoßung von Transplantaten gibt.

## Kommentar

Bei der vorliegenden Cochrane-Übersichtsarbeit handelt es sich um ein relevantes und aktuelles Review. Es ist für uns Urologen v. a. aus zwei Gründen bedeutend. Zum einen ist es für uns essenziell, die Ergebnisse der Nierentransplantation stetig zu verbessern und das Vertrauen in dieses Verfahren in der Bevölkerung weiter zu stärken, insbesondere vor dem Hintergrund des kontrovers diskutierten Organspendegesetzes [1, 2]. Zum anderen gewinnt die Gendermedizin zunehmend an Bedeutung, da sie einen Weg zur individualisierten Medizin und ganzheitlichen Betrachtung der menschlichen Gesundheit darstellt. Gendermedizin hat das Ziel, Bewusstsein für personalisiert Unterschiede zu schärfen und geschlechtsspezifische Zugangsbarrieren zur Gesundheitsversorgung zu beseitigen. Darüber hinaus zielt sie darauf ab, die Diagnose und Behandlung von Krankheiten für alle Geschlechter zu verbessern und diese Ergebnisse in die medizinische Ausbildung einzubeziehen [3]. Andere Autoren gehen mit ihrer Definition noch weiter: Die geschlechtsspezifische Medizin ist der erste Schritt zur personalisierten Medizin und patientenzentrierten Versorgung sowie eine wesentliche Entwicklung, um das Ziel eines ganzheitlichen Ansatzes für Patienten und Erkrankungen als Standard zu erreichen [4]. Weiterhin gibt es Hinweise aus der Literatur, dass das Geschlecht eine Rolle bei der Einhaltung der Einnahme von Immunsuppressiva spielen kann, was wichtig ist, da die Einhaltung bekanntermaßen ein unabhängiger Prädiktor für die Überlebensdauer von Allotransplantaten ist [5]. Zusätzlich haben Querschnitt- und retrospektive Analysen in Nordamerika gezeigt, dass weibliche Transplantationsempfängerinnen im Vergleich zu männlichen Transplantationsempfängern eine höhere Medikamenteneinnahme-Compliance aufweisen [6]. Aus diesen Gründen ist eben auch die Auseinandersetzung mit gendermedizinischen Aspekten in der Nierentransplantation sinnvoll.

Allerdings muss in diesem Kontext ganz klar auf stringente Formulierungen und Definitionen geachtet werden sowie der Unterschied zwischen rein biologischem Geschlecht und anderen geschlechtsspezifischen Aspekten, wie z. B. kulturelle Einstellungen und Verhaltensweisen, klar herausgearbeitet werden. Dies ist bei den inkludierten Studien dieser Übersichtsarbeit nicht immer geschehen und gestaltet sich auch bei der Übersetzung der Cochrane-Arbeit schwierig. Umso wichtiger ist es in zukünftigen Untersuchungen, klare Definitionen und Formulierungen zu verwenden und stringent auf diese Unterschiede hinzuweisen.

Trotz der methodisch hohen Qualität der vorliegenden Cochrane-Arbeit mangelt es den inkludierten Studien oft an dieser Qualität. Zum einen aufgrund des Mangels an klaren Definitionen, wie oben bereits diskutiert. Zum anderen gab es ein hohes Risiko für Verzerrung in vielen inkludierten Studien hinsichtlich der Studienbeteiligung, Ergebnismessung, der statistischen Analyse sowie der ausführlichen, systematischen Berichterstattung aller Ergebnisse. Außerdem hatten einige Studien ein unklares Risiko für Verzerrung bzgl. der Bewertung der Prognosefaktoren. Daher ergibt sich aus dieser systematischen Übersichtsarbeit für die klinische Praxis auch nur ein sehr niedriger bis niedriger Evidenzlevel. Auch eine am 07.12.2025 durchgeführte Update-Suche konnte keine Studien mit den gleichen Einschlusskriterien identifizieren, die den hier vorliegenden Evidenzlevel verändert würden.

Ein letzter Aspekt, der diskutiert werden muss, ist, dass es in der Arbeit keine detaillierte Subgruppenanalyse für die alleinige Nierentransplantation gibt. Diese Daten wären für uns Urologen von besonderem Interesse, da wir vorrangig in die Betreuung dieser Patienten involviert sind, während kombiniert Niere- und Pankreas-Transplantierte vorrangig durch Transplantationschirurgen und Nephrologen betreut werden. Zusätzlich wäre eine Subgruppenanalyse der rein pädiatrischen Population ebenfalls sinnvoll gewesen.

Trotz des geringen Evidenzlevels sehen die Autoren folgende Implikationen für die klinische Praxis: Trotz groß angelegter Registerstudien, die die modifizierenden Auswirkungen von Alter des Empfängers und Geschlecht des Spenders auf den Verlust des Allotransplantats und den Tod belegen, ergab diese Metaanalyse wenig zuverlässige Hinweise darauf, dass das Geschlecht des Empfängers einen Einfluss auf den Verlust des Transplantats nach einer Nieren- bzw. kombinierten Nieren-Pankreas-Transplantation hat. Es gibt keine ausreichenden Belege, um Rückschlüsse auf die Auswirkungen des Geschlechts des Empfängers auf patientenrelevante Ergebnisse nach einer Nierentransplantation zu ziehen. Damit ändert sich für die klinische Praxis zunächst nichts.

Gerade aufgrund diesen geringen Evidenzlevels, der begrenzten klinischen Implikationen und der mangelnden Qualität der inkludierten Studien sind die Implikationen für die Forschung das wichtigste Ergebnis dieser Cochrane-Arbeit. Die Forderungen der Autoren des Reviews sind: (I) Berücksichtigung von biologischen Faktoren, die den geschlechtsspezifischen Unterschieden bei den Transplantationsergebnissen zugrunde liegen könnten; (II) der Einfluss wichtiger Faktoren wie das Geschlecht des Empfängers/Spenders und das Alter des Empfängers/Spenders darf bei der Bewertung der Transplantationsergebnisse nicht außer Acht gelassen werden. Auf diese Weise kann zukünftig vielleicht besser verstanden werden, wie Interventionen wie die Immunsuppressionstherapie an die Merkmale der Empfänger und Spender durchgeführt und angepasst werden können. Ferner sollten Forschungsarbeiten klare Definitionen für Transplantatverlust sowie akute und chronische Abstoßung enthalten sowie geschlechtsspezifische demografische Daten bereitstellen, um Subgruppenanalysen zu ermöglichen. Es sind weiterhin Studien erforderlich, um die Gründe für die bereits beobachteten geschlechtsspezifischen Unterschiede besser zu verstehen, wobei auch, wie bereits dargestellt, das Alter und Geschlecht sowohl von Spender und Empfänger berücksichtigt werden müssen, damit entsprechende Gegenmaßnahmen entwickelt werden können. Geschlecht und Gender sollten in zukünftigen Forschungsarbeiten differenziert und nicht synonym bei der Analyse der prognostischen Relevanz verwendet werden. Das Konzept des Genders wurde als primäre Exposition bisher nur unzureichend berücksichtigt, sodass es bei seiner Verwendung sehr anfällig für Fehlklassifizierungen ist. Außerdem ist eine ausreichende Nachbeobachtungszeit erforderlich, um die Ergebnisse nach der Transplantation zu bewerten und die ätiologischen Prozesse zu untersuchen, die langfristig zur Entstehung dieser Ergebnisse beitragen.

Zusammenfassend muss noch viel qualitativ hochwertige Forschungsarbeit im Bereich Gendermedizin und Nierentransplantation geleistet werden, um Schlussfolgerungen zu treffen bzw. Schlüsse für die klinische Praxis zu ziehen.

## Fazit für die Praxis

- Gendermedizinische Aspekte sind auch in der Nierentransplantation von Bedeutung; in diesem Kontext muss auf klare Definitionen und Begrifflichkeiten geachtet werden.
- Ein interessanter geschlechtsspezifischer Aspekt in der Nierentransplantation scheint die Compliance bei der Einnahme von Immunsuppressiva zu sein, hier könnten weitere Studien sinnvoll sein.
- Es gibt keine ausreichenden Belege, um Rückschlüsse auf die Auswirkungen des Geschlechts des Empfängers/Spenders auf patientenrelevante Ergebnisse nach einer Nierentransplantation zu ziehen.
- Es sind qualitativ hochwertige Studien erforderlich, um die Gründe für die teils beobachteten geschlechtsspezifischen Unterschiede besser zu verstehen, wobei auch das Alter der Empfänger/Spender und das Geschlecht der Empfänger/Spender berücksichtigt werden müssen.
